# Civic Participation Among Latinx and African American Older Adults, an Intersectionality Life-Course Perspective

**DOI:** 10.1093/geroni/igab046.1541

**Published:** 2021-12-17

**Authors:** Laurent Reyes

**Affiliations:** Rutgers University, Rutgers University, New Jersey, United States

## Abstract

Older adults’ civic participation has received considerable attention, but most scholarship has focused on formal volunteerism and voting. The literature shows that rates of voting and volunteering have been consistently lower among African Americans and Latinx older adults compared to their White counterparts. However, little research has explored civic participation in the context of historical structures of inequality that exclude these populations from participating in formal civic activities and continue to do so today. In addition, other civic activities are going unrecognized. To understand civic participation through the lens of Latinx and African American older adults I draw from intersectional life course perspective to contextualize participants’ lived experiences across the life course and within historical and current socio-political space in which they live and participate. Study’s findings could improve conceptualizations and measurements of civic participation for future studies, and inform efforts to support civic participation among these populations.

